# Tickborne Relapsing Fever — United States, 1990–2011

**Published:** 2015-01-30

**Authors:** Joseph D. Forrester, Anne M. Kjemtrup, Curtis L. Fritz, Nicola Marsden-Haug, Janell B. Nichols, Leslie A. Tengelsen, Rick Sowadsky, Emilio DeBess, Paul R. Cieslak, Joli Weiss, Nicole Evert, Paul Ettestad, Chad Smelser, Jonathan Iralu, Randall J. Nett, Elton Mosher, JoDee Summers Baker, Clay Van Houten, Emily Thorp, Aimee L. Geissler, Kiersten Kugeler, Paul Mead

**Affiliations:** 1 Epidemic Intelligence Service, CDC; 2 Division of Vector-Borne Diseases, National Center for Emerging and Zoonotic Infectious Disease, CDC; 3 Division of Communicable Disease Control, California Department of Public Health; 4 Office of Communicable Disease Epidemiology, Washington State Department of Health; 5 Colorado Department of Public Health and Environment; 6 Idaho Department of Health and Welfare; 7 Nevada Division of Public and Behavioral Health; 8 Oregon Department of Human Services; 9 Arizona Department of Health Services; 10 Texas Department of State Health Services; 11 Epidemiology and Response Division, New Mexico Department of Health; 12 Indian Health Service, Gallup Indian Medical Center; 13 Division of State and Local Readiness; 14 Montana Department of Public Health and Human Services; 15 Division of Disease Control and Prevention, Utah Department of Health; 16 Wyoming Department of Health; 17 Career Epidemiology Field Officer

Tickborne relapsing fever (TBRF) is a zoonosis caused by spirochetes of the genus *Borrelia* and transmitted to humans by ticks of the genus *Ornithodoros*. TBRF is endemic in the western United States, predominately in mountainous regions. Clinical illness is characterized by recurrent bouts of fever, headache, and malaise. Although TBRF is usually a mild illness, severe sequelae and death can occur (1*–*4). This report summarizes the epidemiology of 504 TBRF cases reported from 12 western states during 1990–2011. Cases occurred most commonly among males and among persons aged 10–14 and 40–44 years. Most reported infections occurred among nonresident visitors to areas where TBRF is endemic. Clinicians and public health practitioners need to be familiar with current epidemiology and features of TBRF to adequately diagnose and treat patients and recognize that any TBRF case might indicate an ongoing source of potential exposure that needs to be investigated and eliminated.

TBRF is not nationally reportable, and there is no standard case definition. For the purpose of this report, a TBRF case was defined as a clinically compatible illness with laboratory confirmation of infection or a clinically compatible illness epidemiologically linked to a laboratory-confirmed case. In 2011, TBRF was reportable in 12 states: Arizona, California, Colorado, Idaho, Montana, Nevada, New Mexico, North Dakota, Oregon, Texas, Utah, and Washington. TBRF case data for these 12 states for the period 1990–2011 were compiled, along with a single case reported to CDC from Wyoming, yielding 504 cases. Three states accounted for approximately 70% of all reported TBRF cases (California, 33%, Washington, 25%, and Colorado, 11%); the remainder were reported from Idaho, 7%, Nevada, 5%, Oregon, 4%, Arizona, 4%, Texas, 4%, New Mexico, 3%, Montana, 2%, Utah, 2%, and Wyoming, <1% (Figure). No cases were reported from North Dakota. County of residence and county of exposure were known for 325 (64%) cases; 215 (66%) of these cases were reported among nonresident visitors to the counties of exposure (Table).

The median number of cases per year was 20, with a range of 14 in 1993 to 45 in 2002. Median age of patients was 38 years (range = 1–91 years). The age distribution was bimodal, with peaks among persons aged 10–14 years and 40–44 years; 278 (57%) of the patients were male. Race information was not available in the reported data.

Blood smear was indicated as the method of diagnosis for 184 (76%) of 243 cases for which diagnostic information was available. Most (74%) patients had onset of illness during June–September with a peak during July–August (52%). In Texas, cases occurred more frequently (67%) during November–March, and 11 cases (61%) were associated with spelunking.

Most TBRF cases in the United States are caused by *Borrelia hermsii* and transmitted by *Ornithodoros hermsi* ticks. These soft ticks typically live in the nests of rodents such as ground squirrels, tree squirrels, and chipmunks in coniferous forests at elevations between 1,500 and 8,000 feet (457 and 2,438 meters) (5). Soft ticks can acquire TBRF *Borrelia* by feeding on infected rodents, the reservoir hosts; once infected, soft ticks remain infectious for life (6*,*7). The spirochete, which resides in the salivary gland of the soft tick, can be transmitted within 30 seconds of initiation of a blood meal (5). If the rodent reservoir host dies or vacates the nest, soft ticks seek other sources of blood. In locations where rodents and humans are in close proximity (e.g., seasonally occupied lake or mountain cabins infested by rodents), human infections can occur (8*,*9*)*. Unlike hard ticks that embed in the host, soft ticks feed briefly (up to 30 minutes) and typically at night, so most patients are unaware that they have been bitten (5*,*6).

The characteristic clinical feature of TBRF is the occurrence of febrile episodes lasting 3–5 days, with relapses after 5 to 7 days of apparent recovery. This pattern is the result of antigenic variation in spirochete outer surface proteins, temporarily evading the host immune response and allowing spirochete numbers to rebound (5). TBRF is treated with antibiotics, which typically results in cure without sequelae (5). However, complications such as acute respiratory distress syndrome have been described (1*,*3). The risk for transplacental transmission has been documented and pregnant women might be more susceptible to severe complications such as spontaneous abortion, preterm delivery, and perinatal mortality (2*,*4). Clinicians need to consider TBRF in patients with compatible clinical illness and a history of residence in or recent travel to areas that are known foci for TBRF. A diagnosis of TBRF can be confirmed by observation of spirochetes in a blood smear taken during a febrile episode and either stained with Wright-Giemsa stain or examined with dark field microscopy (5*,*10). Testing for serum antibodies is not valuable in the acute setting but might be useful for retrospective identification in convalescent patients (5).

No overall increase or decrease in the annual number of cases reported was observed during the reviewed time period. The bimodal age distribution could reflect differences in clinical manifestations, health care seeking behavior, or exposure to infected ticks. Most cases occurred during the summer months, consistent with arthropod vector biology, reservoir host biology, human outdoor activity, and vacation seasons (5). Outbreaks have been reported among groups of young persons on trips, particularly those sleeping on floors, which might further explain the age distribution (9). Notably, cases in Texas occurred more frequently in winter months and were associated with time spent in caves, which likely represents infection with *Borrelia turicatae,* another species of TBRF *Borrelia* transmitted by *Ornithodoros turicata* ticks (5).

This report is subject to at least two limitations. First, case ascertainment depends upon state-specific practices, and there is no standard surveillance case definition in the 12 western states where TBRF is reportable. Differences in case definitions could lead to ascertainment and reporting bias. Second, TBRF cases likely represent a fraction of the actual incidence because many patients might experience mild, self-limited illness that goes undiagnosed.

Because tick-infested buildings can serve as a source of infection for years, it is important to investigate all TBRF cases to identify the likely location of exposure and guide remediation of rodent and tick infestations. Rodent control alone can increase human risk because any remaining ticks, which can be long-lived, will repeatedly search for alternative hosts. Therefore, it is important to consider tick control in concert with rodent control. Personal preventive practices can include sleeping off the floor and away from walls in rodent-infested buildings and eliminating incentives for rodent residence (e.g., by storing food in tightly sealed containers).* Homeowners in areas where TBRF is endemic can consult with local environmental health specialists and pest removal services on strategies to discourage rodent activity in homes. Persons living in or vacationing in areas where TBRF has been reported need to be aware of the disease and seek medical attention if they develop febrile illness.† Educational outreach would further public health objectives to increase awareness of TBRF prevention measures and clinical signs and symptoms of disease.§

**What is already known on this topic?**

Tickborne relapsing fever (TBRF) is an uncommon cause of febrile illness in the western United States. The most significant risk factor for infection is sleeping in a rodent-infested cabin or house. In 2011, TBRF was reportable in 12 states.

**What is added by this report?**

During 1990–2011, a total of 504 cases of TBRF were reported to CDC. Cases occurred most commonly among males and among persons aged 10–14 and 40–44 years. Three states, California, Washington, and Colorado, accounted for approximately 70% of all reported cases. In counties where most reported TBRF exposures occurred, most infections were among visitors to the counties. Most TBRF infections occur during the summer months during peak arthropod, host, and human activity.

**What are the implications for public health practice?**

Public health practitioners need to be aware of TBRF in locations where it is endemic, and the importance of recognizing and eliminating foci of transmission. Clinicians need to consider TBRF as a cause of febrile illness in visitors to, and persons living in, areas where TBRF is endemic.

## Figures and Tables

**FIGURE:**
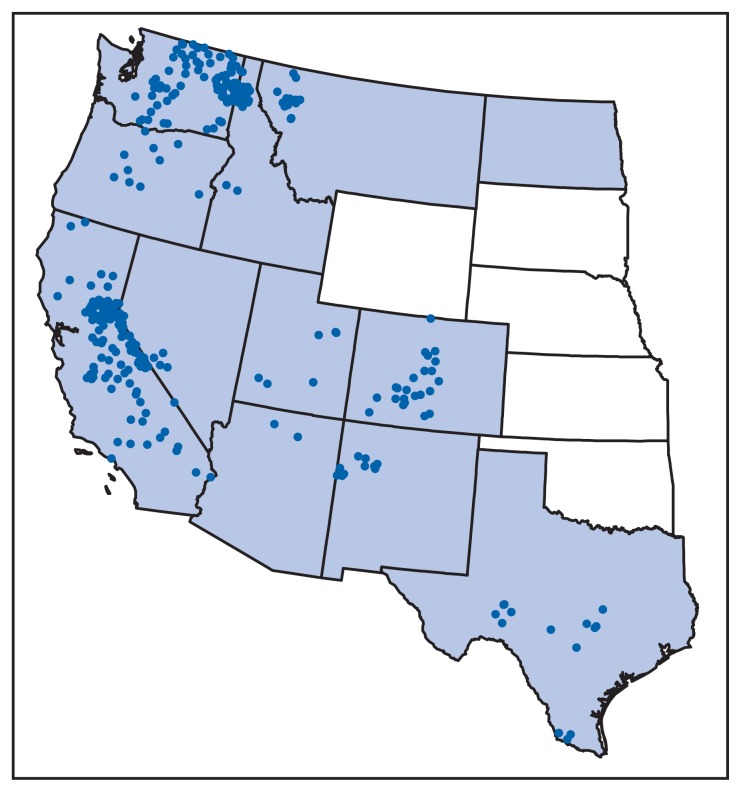
Number of reported cases of tickborne relapsing fever — United States, 1990–2011* * One dot was placed randomly in the county of exposure where known. Clinicians can contact county or state health departments to learn whether tickborne relapsing fever has been reported in a particular county. Shading indicates those states where tickborne relapsing fever was reportable. No cases were reported from North Dakota.

**TABLE:** Ten counties with the greatest numbers of reported cases of tickborne relapsing fever, and the percentage of cases that occurred among nonresidents of the county — United States, 1990–2011

County*	Total no.	Nonresidents	

		No.	(%)
Kootenai, Idaho	**29**	29	(100)
Mono, California	**23**	14	(60.9)
Nevada, California	**20**	15	(75.0)
Spokane, Washington	**20**	1	(5.0)
Okanogan, Washington	**15**	5	(33.3)
Placer, California	**15**	13	(86.7)
El Dorado, California	**14**	10	(71.4)
Lake, Colorado	**13**	8	(61.5)
Fresno, California	**11**	5	(45.5)
McKinley, New Mexico	**11**	7	(63.6)

* Median elevation of the 10 counties was 3,840 feet (range = 1,178–7,562 feet) (1,170 meters [range = 359–2,305 meters]).

## Footnotes

* Additional information available at http://www.cdc.gov/relapsing-fever/prevention.

† Additional information available at http://www.cdc.gov/relapsing-fever/symptoms.

§ Additional information available at http://www.cdc.gov/relapsing-fever/clinicians.
